# An Evaluation of Strategies Used to Maximize Intervention Fidelity in a Randomized Controlled Trial of a Sexual Assault Resistance Program for University Women

**DOI:** 10.1007/s11121-021-01239-2

**Published:** 2021-04-17

**Authors:** Karen L. Hobden, Wilfreda E. Thurston, Gail L. McVey, Charlene Y. Senn

**Affiliations:** 1 Department of Psychology, University of Windsor, 401 Sunset Avenue, Windsor, ON Canada; 2 Department of Community Health Sciences, University of Calgary, Calgary, Canada; 3 Toronto General Hospital, University Health Network, Toronto, Canada; 4 Department of Women’s and Gender Studies, University of Windsor, Windsor, Canada

**Keywords:** Intervention fidelity, Randomized controlled trial, Sexual assault prevention

## Abstract

In this paper, we describe and evaluate the strategies used to maximize intervention fidelity in a randomized controlled trial to examine the efficacy of a sexual assault resistance intervention. The EAAA program was based on the best available theory and evidence on how women can successfully resist sexual coercion from male acquaintances. Extensive protocols for hiring, training, and supervising facilitators were established a priori. Detailed intervention manuals were developed that clearly described program goals, learning objectives, core elements, troubleshooting tips, sections that must be delivered verbatim, adaptations that could be made if necessary, and the ideal and minimum dose. Program sessions were audio-recorded, and a subsample of recordings were scored for adherence to the manuals using detailed Intervention Fidelity Checklists (IFC) developed specifically for this research. The Gearing et al. (2011) Comprehensive Intervention Fidelity Guide (CFIG) was employed retrospectively to provide objectivity to our analysis and help identify what we did well and what we could have done better. The SARE (Sexual Assault Resistance Education) Trial received high scores (38 out of 44 (86%) from each of the first two authors on the CFIG, suggesting a high level of intervention fidelity. Although a potential for bias on the part of the two raters was an obvious limitation, as was our neglection to include measures of implementation receipt, which Gearing et al. (2011) recommended, our analysis underscores the utility in employing methods recommended to enhance intervention fidelity when developing and evaluating evidence-based interventions.

Sexual assault is a major public health issue, underscoring the need for preventative interventions (DeGue et al., 2014). The burden of trauma and related symptoms is costly to victims of sexual violence (Basile et al., 2006; Day, 1995). In addition, there are related health care costs associated with treating co-morbid conditions resulting from sexual assault (Deliramich & Gray, 2008; Perilloux et al., 2012; Young et al., 2011) as well as numerous social costs (Peterson et al., 2017; Post et al., 2002).

The Enhanced Assess, Acknowledge, Act (EAAA) program is a small group, evidence-based, sexual assault resistance education program delivered to first year undergraduate women by pairs of well-trained peer educators. As shown in a randomized controlled trial (RCT), the EAAA program fills an important service delivery gap by reducing the incidence of rape/sexual assault among women on university campuses (Senn et al., 2015, 2017). First year female undergraduate students who received EAAA were 46% less likely to experience completed rape (*p* = 0.021) and 63% less likely to experience attempted rape (*p* = 0.001) over the next year compared to their female counterparts in the control arm. The incidence of attempted coercion and nonconsensual sexual contact were also reduced by 36% (*p* = 0.001) and 34% (*p* = 0.001), respectively (Senn et al., 2015, 2017). Further, participation in EAAA increased women’s perception of personal risk, self-defense self-efficacy, knowledge of effective (forceful verbal and physical) resistance strategies, and decreased rape myth acceptance and women blaming over a 2-year follow-up period, helping to prevent re-occurrences of rape (Senn et al., 2017). EAAA provides women with the best available strategies for resisting sexual coercion from known men and the confidence to use those strategies. It also reinforces their knowledge that the perpetrator is always responsible for sexual violence and the victim blameless.

The manualized nature of EAAA together with its high efficacy makes it a desirable, accessible, and cost-effective intervention to scale up across postsecondary institutions. In order to replicate high levels of efficacy, attention must be paid to rigorous implementation protocols to ensure fidelity to the intervention model (Pinnock, 2015; Pinnock et al., 2017). The present study outlines in detail the methods used by the EAAA intervention team to measure, monitor, and maximize the fidelity and corresponding efficacy of the intervention throughout all phases of the research. To help us codify the extent to which we were successful in our efforts, we retrospectively utilized a scoring mechanism developed by Gearing et al. (2011). Our findings have implications for enhancing the uptake of implementation practices necessary to yield positive results from this evidence-based sexual assault resistance intervention.

Gearing et al. (2011) reviewed three decades of medical and psychosocial research on how best to assess and optimize intervention fidelity. The result was a detailed framework designed to assist future researchers to maximize intervention fidelity during the development, planning, and implementation of interventions. The authors identified four major phases in which fidelity should be assessed: (1) Intervention Design, (2) Intervention Training, (3) Monitoring of Intervention Delivery, and (4) Monitoring of Intervention Receipt. Intervention Design deals with the theoretical underpinnings and goals of the intervention, the development of manuals for delivering it (including the environment, mode of delivery, possible adaptations, and maximum and minimum dose). During this phase, the qualifications, characteristics, and training standards for interventionists, trainers, and supervisors should be identified. The design of a possible evaluation trial should also be considered at this time including the eligibility criteria for research participants, the measures to use at each stage, and the identification of possible threats to internal and external validity. The Intervention Training phase concerns the development of protocols for training and supervising interventionists. Protocols should also be developed for identifying drift in intervention delivery and for reviewing and updating the intervention when required. Monitoring of Intervention Delivery deals with administration protocols developed and measures selected to assess intervention fidelity, including the competence of interventionists and their adherence to prescribed intervention delivery. Deviation from intended delivery can reduce the effectiveness of an intervention and make it difficult to interpret results of research designed to test its efficacy. In the fourth and final component, Monitoring of Intervention Receipt, investigators assess not only whether a participant was present when an intervention was delivered, but whether they understood, were engaged, and complied with the intervention’s content. To assess this aspect of intervention fidelity, the authors recommended measuring participants’ knowledge, comprehension, and compliance with the intervention before and after its delivery. Interestingly, in their review, Gearing et al. (2011) found that investigators placed the greatest emphasis on Monitoring Intervention Delivery, with less focus placed on Intervention Design and Interventionist Training, and little or no attention paid to Intervention Receipt.

Finally, the authors developed a scoring procedure, the Comprehensive Intervention Fidelity Guide (CIFG), to assist investigators in assessing intervention fidelity in their own research. In the current paper, we used the Gearing et al. (2011) model as a retrospective framework for describing our efforts to maximize intervention fidelity in the SARE (Sexual Assault Resistance Education) Trial. We also used the CIFG to score our efforts. Below we describe the explicit strategies we used to maximize fidelity organized using Gearing’s four phases. Later, in the Methods section, we provide detail on the measures and/or data used for each phase.

## Our Strategies Used to Maximize Implementation Fidelity

### Intervention Design

#### A Theory Driven, Evidence-Based Intervention.

EAAA is anchored in feminist and social psychological theory and supported by empirical evidence shown to reduce women’s risk of sexual violence from male acquaintances. It was developed, pilot tested, and revised over several years. The theoretical underpinnings have been described extensively elsewhere (Senn, 2011; Senn et al., 2013).

The goals of the program are to assist women to (1) more quickly identify potential or actual risk cues for sexual violence in social settings and/or in men’s behaviour, (2) trust their judgement and perceptions, (3) overcome their emotional barriers to risk detection and resistance, and (4) increase their confidence in their ability to choose from and successfully employ an array of effective strategies for resisting sexual assault from male acquaintances. The program emphasizes that the perpetrator is always to blame for sexual violence, does not promote any one preventative strategy, and underscores that women are always the best judge of how to respond to sexual violence.

#### A Well Articulated, Manualized Program with Intervention Delivery Guidelines.

A well-articulated, manualized program was developed to guide effective implementation. The program format consists of scripted mini-lectures, games, activities, small and large group discussion topics, and role-playing scenarios. A program overview guide was developed to provide facilitators with general guidelines for program delivery, the rationale for the setting and timing of delivery, and ways to manage time constraints without compromising content. Within the manual for each unit, learning outcomes and verbatim scripts were provided, with key points listed for the few unscripted sections. Instructions on how to troubleshoot potential challenging situations were included to help optimize facilitation. Tangible materials such as PowerPoint presentations, posters, scenario cards, and audio and visual recordings were included in the EAAA Program Kit.

### Interventionist Training

#### Implementation Training and Coaching.

EAAA is delivered by facilitators working in pairs with the lead facilitator taking a more dominant role within each program offering. All facilitators were required to learn both the lead and secondary roles as part of their training and were assigned as the lead facilitator for half the programs they facilitated. Newly hired facilitators completed a comprehensive reading list on the theory and evidence-base behind the intervention’s content and had weekly meetings (4 times) with their supervisor to discuss the readings and have questions answered. Only after the readings were completed were facilitators given the manuals and the rest of the EAAA Program Kit and began practicing program delivery alone and with their co-facilitators. An 8-day training program was then provided by the program developer for all facilitators together at a central site in late August prior to the beginning of data collection. At least one co-investigator from each site also attended this training to buttress their supervision of facilitators in delivering EAAA. The training consisted of didactic learning sessions (2 days), critiqued, mock delivery of the 3 non-self-defense units of the program to student audiences (3 days), and 3 days of Wen-Do self-defence instruction (a 2-day Basic Course followed by 1 day of individualized instruction). Mock delivery with critique provided facilitators with the opportunity for behavioural rehearsal, a best practice for maximizing training success and intervention fidelity according to researchers in intervention science (Beidas et al., 2014; Cross et al., 2011). The program developer then travelled to each research site to assist the co-investigators to supervise and critique an additional 1-day mock delivery of the self-defense unit (again with a student audience) once facilitators had time to practice.

#### Practice Based Learning and Skill Building.

Between annual trainings, facilitators were required to deliver dress rehearsals of all four EAAA units (using all program materials and equipment) under the supervision of co-investigator experts at each of the participating universities. Full dress rehearsals in front of a mock audience were only required in the winter semester as facilitators would have just practiced in front of a mock audience at the August training. The self-defense portion of EAAA is based on Wen-Do Women’s Self-Defence. Because it takes considerable skill and practice to become a Wen-Do instructor (requiring years of training), scale up would have been impossible if facilitators needed to be certified Wen-Do instructors. Instead, considerable emphasis was placed on the skills facilitators needed to accurately deliver the subset of self-defense techniques covered in this unit. Finally, facilitators were instructed to maintain a journal detailing their thoughts and feelings after each EAAA session. This exercise was intended to help facilitators reflect on their delivery of EAAA and develop greater self-awareness of their strengths and challenges.

#### Refresher Training.

Prior to the second year of data collection, returning facilitators reviewed key background readings (a subset of those read before the original training) and attended a slightly shorter (6 day), in-person, refresher training together at the central site. This training consisted of 1 day of didactic instruction, practice sessions with critiques from the developer (3 days), and a repeat of the 2-day Basic Wen-Do Course. The developer subsequently travelled to each research site to assist with the critique of facilitators’ final (self-defense unit) practice session. New facilitators attended the refresher training with returning facilitators but were required to complete the full list of readings and three days of self-defense instruction. They were assigned secondary facilitator roles in their first facilitations.

### Monitoring Intervention Delivery

#### Self-Monitoring of Adherence.

Facilitators were instructed to complete a Fidelity Issues Form (FIF) after any program session where their delivery deviated from the corresponding manuals. They were expected to bring any completed FIFs along with their journal notes to their supervisory meetings with the program developer. The notes were not examined but rather acted as prompts to memory for facilitators for discussions of adherence.

#### Supervisory Meetings.

The monitoring of the facilitators’ adherence to the program content was accomplished through regular supervisory meetings led by the program developer and attended by a co-investigator at one site and a site coordinator at the second site. All three facilitators at each site were required to attend these meetings following delivery of each weekly or weekend program session thereby providing an opportunity for the facilitators who had just delivered EAAA to debrief together and address any issues that arose, but also allowing the third facilitator to benefit from any lessons learned by her colleagues. During these meetings, after inquiring as to facilitators’ impressions of the most recent session, the program developer and facilitators would examine the FIFs for issues emerging from the session backed up by facilitators’ sharing of pertinent issues raised in their self-reflection journals. Finally, they would page through the corresponding manual together as a means of jogging facilitators’ memory for further deviations from intervention protocols.

#### Audio Recordings of Program Sessions.

Program sessions were recorded (with permission of participants) and a subsample of these recordings were scored to assess facilitators’ adherence to the corresponding program manual.

### Monitoring Intervention Receipt

#### Attendance.

Participant attendance was taken at each session as a measure of intervention receipt.

## Methods

### Participants

#### Program Facilitators

Twelve female graduate and senior undergraduate students from Psychology, Sociology and Social Work under the age of 30 years (with the exception of two) were hired and trained as facilitators across the three university sites, with priority given to those who could commit to the initiative for 2 years. Because the program is delivered in pairs, three facilitators were hired at each site and pairs rotated so that there was always a third facilitator available as a backup if needed. A behavioural interview protocol was developed for screening potential candidates for the facilitator role with requisite experience and preferred skills established from the outset. Of the 9 facilitators initially hired, 6 stayed on for the full 2 years of data collection (67%). Three replacement facilitators were hired and trained in the summer before the second year of data collection.

#### Program Implementation Supervisory and Monitoring Team

The facilitators were supervised directly (in person or remotely) by the program developer, and one or more co-investigator at each of the two sites where the developer was not based, all of whom were experts in violence against women. The clinical trial manager, who had expertise in managing multi-site research, was responsible for ensuring facilitators were aware of their duties regarding the research and intervention fidelity (e.g., procedures for audio recording program sessions, completing Fidelity Issues Forms).

### Measures

#### Fidelity Issues Form

A Fidelity Issues Form (FIF) was developed to capture facilitators’ immediate, post-session, self-reports of deviations from the manualized program. This form had a yes/no response format, with additional open-ended questions to solicit general feedback about the quality of the session delivery (e.g., Was the content delivered as expected, if not, why?). Facilitators were directed to complete an FIF whenever delivery of a program session diverged in any way from its description in the manuals. In fact, they were encouraged to complete one after every session regardless of whether they thought it was needed to ensure that all deviations were reported. Facilitators were also instructed to bring completed FIFs to their supervisory meetings with the program developer for review.

#### Intervention Fidelity Checklists

Providing an objective way to monitor intervention delivery is essential for assessing intervention fidelity. To this end, detailed Intervention Fidelity Checklists (IFCs) and instructions for scoring were developed prior to the beginning of data collection that aligned with the manualized program content. The IFCs were used to score a randomly selected subsample of audio recordings of program sessions. Facilitators were trained to audio record each EAAA session they delivered. A randomly selected subsample (25% stratified by site and lead facilitator) of these audio recordings were scored for intervention fidelity using the IFCs described above.

#### Attendance Forms

Measurement of participant attendance at each of the four program sessions was essential for monitoring intervention delivery and receipt. EAAA attendance was recorded by the facilitators who were instructed to note participants who arrived late, left early, or missed sessions. Full attendance at any one session was defined as missing no more than 30 min of the 3-h session (to allow for arriving late, leaving early, taking washroom breaks, etc.).

#### Comprehensive Intervention Fidelity Guide

The Gearing et al. (2011) 22-item Comprehensive Intervention Fidelity Guide was used to assess intervention fidelity in the RCT. The checklist captures intervention fidelity across four phases of intervention development and evaluation: (1) Intervention Design, (2) Intervention Training, (3) Monitoring Delivery of the Intervention, and (4) Monitoring Receipt of the Intervention. The specifics of the checklist include items that assess whether or not the following procedures were in place: (a) protocols to guide the assessment and measurement of intervention fidelity, (b) an explication of the manner by which fidelity is assessed and measured, (c) the procedures used to promote consistency in the application of fidelity through all stages of implementation, (d) collection of intervention fidelity data and corrective changes made to the design or delivery to maximize fidelity, and (e) the recording of internal or external variables that can negatively impact intervention fidelity. Each item or element on the checklist is scored on a three-point scale, ranging from 0 to 2 with higher scores indicating greater fidelity (Absent/Minimal = 0, Moderate = 1, or Extensive = 2)

### Procedures

Audio recordings of program sessions were scored by two independent raters using the corresponding IFCs. In total, 192 sessions were delivered at the three sites across the 2 years of participant enrolment in the trial. Fifty-two session recordings were randomly selected for fidelity scoring stratified by site and lead facilitator. Due to technical issues, one session was not recorded at all and one was only partially recorded, neither of which could be included in our selection. Because of the stratification, the number of recordings selected is larger than would be expected if we simply selected 25% of all sessions delivered. Most of the recordings were scored after data collection was complete. However, at the request of the facilitators, a sample of session recordings (i.e., three from each site) were scored after the first year of data collection in order to provide facilitators with feedback on their delivery of EAAA.

Selected session recordings were initially scored by the clinical trial manager. After data collection was complete, a second rater (trained by the trial manager) scored a subsample of 28 (50%, also randomly selected) of those already scored. IFC scores were converted to percentages for ease of comparison across units. The two raters compared scores on the IFCs and resolved any disagreements until their interrater reliability attained 90%.

Each of the first two authors independently reviewed the strategies used to maximize intervention fidelity in the EAAA trial and scored our efforts using the Gearing et al. (2011) CIFG checklist.

## Results

### Intervention Delivery

#### Fidelity Issues Form

We expected that facilitators would complete an FIF after most (i.e., at least 90% or 174) of the 192 program sessions delivered. However, it became apparent during the early stage of data collection that facilitators were reluctant to complete an FIF thinking that doing so would reflect badly on them. Compliance increased slightly once it was explained to them that because at least some deviation from protocol is unavoidable, they were expected to complete an FIF most if not all of the time and that these reports should include any deviation from standard delivery no matter how slight even if it had a positive effect (e.g., a participant shared an uplifting story where they or someone they knew fought off a potential attacker). Nevertheless, only 117 FIFs were received by the Coordinating Centre (67% of the 174 expected). In addition, during the regularly scheduled supervisory meetings early in the second year, it was discovered that one activity in the fourth unit (Relationships and Sexuality) was carried out incorrectly by a facilitator (and her two co-facilitators) at one site throughout most of the first full year of program delivery, but she/they had not noted the deviation on a FIF. Finally, in scoring the audio recordings for intervention fidelity, the clinical trial manager discovered five additional instances where delivery of a program session had obviously deviated from intended delivery, but the facilitators had neglected to complete an FIF.

#### Intervention Fidelity Checklists

Maximum scores on the IFCs ranged from 120 to 293 depending on the unit, with higher scores indicating greater adherence to the delivery instructions and content of the manuals. These scores were converted to a percentage of unit that was delivered correctly. The mean IFC score across the four units was 94.2% with a range of 80.9% and 100% for the first rater, and 93.8% with a range of 88.0% and 100% for the second rater.

### Intervention Receipt

#### Attendance

A majority of program participants (76%) attended all four program sessions in their entirety, with 91% attending at least three session, 94% attending at least two, and 97% attending at least one complete session. Two participants who were assigned to the treatment arm left immediately after randomization without attending any of the program. Three participants attended only part of the first session but missed the remaining three. All five of these participants remained in the study.

#### Intervention Fidelity Across All Four Phases

The Comprehensive Intervention Fidelity Guide (CIFG) was used to assess our efforts in maximizing intervention fidelity across all four phases of intervention research identified by Gearing et al. (2011). The methods used in the RCT earned a score of 38 out of a possible 44 (86%) on the CIFG from each of the first two authors. The sub-scores for each component of the intervention fidelity methods are presented in Table 1 along with a description of what data or information were used to obtain this score. As the table shows, the two raters did not completely agree on the scoring of each component even though the final scores were the same.

**Table 1:** EAAA Trial Scores on the Gearing et al. (2011) Comprehensive Intervention Fidelity Guide

Gearing et al. (2011) Recommendations	Data / information Used to Score EAAA Trial	Rater 1	Rater 2
Intervention Design			
1 Framework – strong theoretical orientation, clearly articulated program goals, identification of ideal environment and mode of delivery, inclusion and exclusion criteria for study participants, education and experience for interventionists, and team structure	The intervention was grounded in the best available theory, evidence, and practices. Learning objectives and goals for each session were outlined in the manuals. Inclusion / exclusion criteria for participants were established a priori. Facilitators’ education, experience, and other characteristics required or preferred were set a priori. A strong research team was established to conduct the RCT including an experienced trial manager, local investigators with VAW background, as well as a site coordinator, 2–3 research assistants, and 3 facilitators at each site	2	2
2 Establish Training Protocols	Extensive training protocols were developed consisting of: • pretraining readings, • a training schedule, • PowerPoint slides with notes for didactic sessions, dress rehearsals of each (behavioural rehearsal), critique, and feedback	2	1
3 Manual – including a program model, well-defined objectives, procedures, outcomes, the ideal and minimum dose, timing of delivery, core elements, troubleshooting, and cultural considerations	Detailed manuals clearly described: • program goals, • learning objectives, • core elements, • troubleshooting tips, • sections that must be delivered verbatim, • adaptations that can be made if necessary, • ideal and minimum dose Feedback on program content was obtained from women from diverse ethnic and cultural backgrounds. Manuals are updated regularly to reflect changes in research and theory as well as societal trends, although during the RCT modifications to the manuals were kept to a minimum	2	2
Interventionist Training			
4 Training Protocols	Training protocols included: • pre-training readings, regular facilitator meetings to discuss the readings, • 2 days of didactic sessions, • 4 days of dress rehearsals of program sessions with audience members, • 3 days of Wen-Do instruction, • Annual refresher training Facilitators were trained and given practice on responding to participants’ questions or comments, dealing with women blaming, and general group facilitation skills (e.g., encouraging participation, dealing with quiet / chatty groups)	2	2
5 Supervision Protocols	Detailed protocols for supervising facilitators were developed that included: • meeting with each team of facilitators after each program session, • supervision using facilitator reflections (including in their journals), FIFs, and paging through intervention manuals to increase points for discussion concerning delivery of activities and scripts, participants’ responses, etc This allowed for identification and remediation of gaps in facilitators’ intervention delivery skills	2	2
6 Maintenance Protocols	After training, but before delivering EAAA, facilitators were required to go through a second round of dress rehearsals of three of the four units (using all equipment and program materials) at their own site under the supervision of the local co-investigator (CI) and/or site coordinator (SC) to ensure they demonstrated competence. In semesters when there was no training, facilitators were again required to do full dress rehearsals of each program unit under the supervision of the CI and/or SC in the presence of a mock audience. Veteran facilitators were required to complete a refresher training the year following initial training	2	2
7 Threats – identification of factors that might threaten the internal or external validity of training such as intervention complexity (internal) or contamination (external)	Although the scripted nature of the intervention minimized threats to intervention fidelity created by its length and complexity, providing regular (rather than annual) feedback to facilitators from reviewed audio recordings of program sessions would have enhanced this effort	1	1
8 Measurements – selection of instruments to assess transfer of training to interventionists (e.g., a pre-and post training measures of knowledge, competence, and confidence)	IFC scores of session audio recordings constituted the only measure of transfer of training to facilitators. We did not include a pre-post measure of facilitator knowledge, confidence, or competence	1	1
Monitoring Intervention Delivery			
9 Differentiation	Core program elements were identified in the intervention manuals as were activities or sections of that could be modified or eliminated altogether. Behaviors facilitators should avoid or minimize were also described in the manuals	2	2
10 Intervention Components	In addition to core elements, the minimum dose required for the program to be effective was described in the manuals, including a discussion of which sessions can and cannot be missed and why	2	2
11 Interventionist Behaviors	Facilitator behaviors were monitored through regular meetings with the program developer (described above), and detailed scoring of audio recordings of programs sessions based on inclusion of content contained in program manuals	2	2
12 Rater Standards	During training, raters were required to attain interrater reliability of 90%	2	1
13 Interventionist Competence	Monitoring of facilitator competence was accomplished through use of the IFCs to score audio recordings of program sessions and through regular supervisory meetings between the facilitators and the program developer where facilitators’ self-report on the FIFs of deviations from standard intervention delivery and journal notes were discussed	2	2
14 Monitoring Drift – monitoring the extent to which interventionists drift away from the preordained standard in their delivery of the intervention	In order to minimize drift and maximize consistency in facilitators’ skills, all facilitators were required to deliver full dress rehearsals of each program session in front of a mock audience during each semester of data collection. In the semester in which facilitators were trained, these dress rehearsals were supervised by PI. In subsequent semesters, they were supervised by the CI and/or SI	2	2
15 Corrective Feedback	Facilitators were given corrective feedback in their regular supervisory meetings with the program developer. Meetings were scheduled after each program session (for weekday programs) or each complete program (for weekend programs)	2	2
16 Threats –factors or variables that might impact the internal and external validity of the delivery of the intervention such as participant motivation (internal) or interventionist attrition (external)	Participant motivation appeared to be high, as indicated in the low rate of attrition across program sessions. Turn over among facilitators was low suggesting their motivation to deliver the program was also high	1	2
17 Measurements – instruments used to measure intervention delivery (e.g., independent observation of intervention delivery)	Intervention delivery was monitored through the review and scoring of 25% of the audio recorded program sessions	2	2
Monitoring Intervention Receipt			
18 Protocols for Dose Received	Protocols were given to facilitators for taking attendance. Participants were considered to have attended an entire session if they missed no more than 30 min of the 3-h session	1	2
19 Participant Comprehension	Measures administered to participants one week after program delivery and again every six months for up to two years were intended as measures of program effectiveness but could be considered measures of intervention receipt (although they were not selected as such)	1	2
20 Participant Adherence – the extent to which participants comply with the intervention	As with participant comprehension, the measures used to assess program effectiveness suggested that participants adhered to the lessons learned in the program. The high participant attendance and low incidence of disruptive behavior on the part of participants (both of which were monitored in attendance records and FIFs and reviewed in supervision meetings) are also suggestive of participant adherence	2	2
21 Threats – factors or variables that might threaten the internal or external validity of participants receipt of the intervention such as participant anger/hostility towards it (internal) or difficulty scheduling intervention sessions (external)	Threats to internal and external validity were not measured specifically. Possible barriers to participation in the intervention were not assessed	1	0
22 Measurements – the instruments selected to measure participants enthusiasm for, understanding, and memory of the intervention (e.g., pre-and-post intervention knowledge and comprehension measures)	Outcome measures, which could also be conceptualized as measures of intervention receipt, were selected for their strong psychometric properties	2	2
	TOTAL Scores	38	38

Following the Gearing et al. (2011) guidelines, each element necessary for Intervention Fidelity was scored on a 3 scale (Absent/Minimal 0, Moderate 1, Extensive 2)

## Discussion

The findings from this study revealed that the SARE Trial led by Senn et al. (2015, 2017) showed high intervention fidelity overall, particularly in intervention design and interventionist training. Multiple measures used in the RCT converged to support the latter conclusion and scores on the Gearing et al. (2011) CIFG buttress this conclusion. In designing the intervention, careful attention was given to creating a theoretically driven, empirically based program supported by highly scripted intervention manuals and an implementation guide. Detailed and extensive training protocols were developed and implemented. New facilitators underwent 9 days of intensive training including dress rehearsals of program sessions critiqued by the developer in the presence of a mock audience. As mentioned previously, providing interventionists with the opportunity for behavioral rehearsal has been identified as a best practice in intervention science for promoting successful training and maximizing intervention fidelity (Beidas et al., 2014, Cross et al., 2011). Intervention delivery was intentionally monitored, and fidelity measured. Self-reported adherence checks were employed in the form of Fidelity Issues Forms (FIFs). Intervention sessions were audio-recorded, and a subsample of these recordings scored for intervention fidelity by two independent raters. Facilitators’ willingness to comply with intervention fidelity measures was moderate to high as indicated by the number of FIFs completed (67%) and program sessions recorded (99%).

Facilitator training was supplemented with session by session supervision with the developer. These meetings resulted in prompts to carry out protocol adherence or correct protocol deviations. They also provided the opportunity for facilitators to create and participate in a community of practice of sorts that prompted peer to peer support and sharing of lessons learned, all of which helped to enhance their competencies in the facilitation process. There were other benefits to supplementing self-report adherence checks with live supervisory meetings including the discovery that one activity was carried out incorrectly by a facilitator at one site throughout the first year of the trial. Perfection in intervention delivery is an elusive outcome. If a facilitator does not see their mistake, they will not record or discuss it. Multiple checks on intervention fidelity are, therefore, recommended. The highly scripted nature of this intervention, the inclusion of troubleshooting tips, and the extensive training and ongoing supervision likely contributed to the high intervention fidelity scores obtained overall.

It is worth noting that in the SARE Trial, all four components of intervention fidelity recommended by Gearing et al. (2011) were assessed, a rarity in the literature. The program was subjected to evaluation from the beginning of its development, demonstrating that an early commitment to intervention fidelity was made by the team.

Despite having multiple outcome measures, participants were not asked to complete assessments of their understanding or recollection of what they learned in a way that was specifically designed to assess this learning. They were asked, at each follow-up time point, what if any strategies from EAAA they subsequently put into practice to resist sexual coercion (the results from this research will be published in a forthcoming paper), but these were intended as outcome measures rather than measures of implementation receipt. We recognize that including measures of implementation receipt would have been beneficial from the standpoint of assessing intervention fidelity; however, any additional assessment steps would have increased the already high research burden for participants. Balancing the benefits of implementation measurement with the risk of participant attrition due to burden are key features of consideration when designing longitudinal intervention research. Our ability to retain research participants over time (95% retention rate across 12 months; Senn et al., 2015) suggests this was a good compromise.

### Limitations of the Study

The main limitation of this study is that the data and other documents supporting the extent to which intervention fidelity was maximized were compiled and reviewed retrospectively by the first two authors on this paper, both of whom were involved in the original RCT, which may have biased their ratings on the CIFG. The CIFG offers some objectivity to assessment of intervention fidelity, as shown in the range of scores among raters. Inclusion of the third author, who was not involved in the original trial, helped to mitigate but not eliminate potential biases of interpretation of all results. Fortunately, the results concerning attendance collected by several facilitators and the independently rated IFCs are consistent with the scores on the CIFG.

Further, additional steps could have been taken to increase intervention fidelity in the EAAA trial. As is commonly reported in other implementation research (Gearing et al., 2011), there were no objective measures to assess the impact of training on the facilitators’ knowledge or skill level, although the program developer informally monitored the impact of training on facilitators’ competence during the initial training and throughout the trial. Having recordings of the program sessions, a sample of which could be rated on an Intervention Fidelity Checklist was a reasonable replacement. In addition, because facilitators worked in pairs, it is possible that learning deficits in one or the other were compensated for by her colleague.

It would have been helpful to include measures of facilitators’ engagement with and enthusiasm for the program. Some indication of facilitators’ compliance can be gleaned from the number of audio recordings collected and FIFs received, although the number of FIFs completed was lower than expected. Even after we explained to facilitators that the FIFs were not intended to be punitive as variations in program delivery are inevitable, expected, and at times unavoidable (e.g., resulting from a fire alarm or power disruption), our reassurances did little to improve facilitators’ compliance. Further, although facilitators were directed to maintain journal notes after each program session they facilitated and to bring these notes to their supervisory meetings, in an effort to protect their privacy, these journals were never collected. As a result, the extent to which facilitators complied with this direction cannot be determined. Based on our observations, facilitators appeared to be compliant with and attentive to the procedures around recording of program sessions and the transfer of these recordings to the Co-ordinating Centre. They also seemed eager for and receptive to feedback on their delivery of EAAA from the developer, although there were occasions when facilitators expressed frustration with the level of critique they received during dress rehearsals. In fact, some veteran facilitators (i.e., those returning for a second year of program delivery) expressed reluctance and dismay at having to continue to perform critiqued dress rehearsals in front of the program developer or their site coordinator/investigator, arguing that because of their level of expertise these requirements were unnecessary and overly onerous. Their overconfidence was always made apparent during these rehearsals when at least one, if not several, activities these facilitators were certain they had mastered completely were delivered incorrectly after the 6-month hiatus between the end of program delivery in the previous academic year and the fall semester of the subsequent year. Perceptions aside, it is likely that facilitators varied in their willingness to comply with procedures put in place to maximize fidelity, but without an objective indicator of compliance we cannot say for certain.

### Future Directions

The findings stemming from assessment of intervention fidelity can aid in more nuanced interpretation of the results of outcome or efficacy research (Greenberg & Barnow, 2014; Rychetnik et al., 2002) particularly when implementation is conducted across multiple sites, as was the case with the EAAA trial (Greenberg & Barnow, 2014; Ofek, 2016). Applying the components of the Gearing et al. (2011) intervention fidelity framework to RCTs such as the one discussed here can help enhance the quality of the design and provide insight into the interpretation of the outcomes.

Future research could examine implementation in a more naturally occurring setting than an RCT where there may be greater variance in levels of intervention fidelity, allowing for the identification of a threshold of fidelity monitoring that yields positive outcomes in delivery of the intervention. This is worthwhile to explore given the resource intense nature of fidelity monitoring. Of note, innovative methods to capture and rate fidelity are beginning to be studied. For example, Caperton et al. (2018) compared the fidelity monitoring of partial sessions (of a psychosocial intervention) with full sessions against interrater agreement and found that approximately a third of a session had sufficient agreement to approach interrater levels. The results from such implementation research measurements can inform the development of feasible and cost-effective ways to modify intervention delivery and help ensure scale up. The findings from the present study show how methods that are recommended to enhance intervention fidelity optimized the efficacy of an evidence-based sexual assault resistance education program.
